# Case Report on a Rare Intraoperative Finding of Ectopic Liver Tissue Attached to Gallbladder Wall during Laparoscopic Cholecystectomy

**DOI:** 10.1155/2019/1046909

**Published:** 2019-10-15

**Authors:** Mohamed Isa, Hussain Al-Mulla, Amal Al-Rayes, Raed Al-Marzooq, Roopa Arora

**Affiliations:** Surgical Department, Salmaniya Medical Complex, P.O. Box 12, Bahrain

## Abstract

**Introduction:**

Ectopic liver is a rare finding (Corsy, 1922; Kubota et al., 2007) that is usually discovered intraoperatively or during an autopsy (Bassis and Izenstark, 1956). Preoperative diagnosis of ectopic liver is also uncommon. The most common site of ectopic liver is on the gall bladder, although there are reports of other sites such as the adrenal glands and esophagus. The management of ectopic liver is en-bloc resection due to the high risk of hepatocellular carcinoma.

**Case Presentation:**

We describe the case of a 42-year-old female who presented with recurrent abdominal pain. She was found to have a smooth fragment of a reddish brown tissue attached to the anterior surface of the gallbladder during an elective laparoscopic cholecystectomy. The tissue was removed with the gallbladder, and histopathology showed normal ectopic liver tissue.

**Conclusion:**

Due to the possibility of malignant transformation into hepatocellular carcinoma, en-bloc resection is the choice of management.

## 1. Introduction

Ectopic liver tissue is a rare occurrence [1, 2] in which liver tissue is placed outside the liver without any hepatic connection [3]. It is often discovered incidentally during laparoscopy, laparotomy, or during an autopsy [4]. Although rare, it has nonetheless been reported in several case reports [4–9]. Ectopic liver has been found above and below the diaphragm, but the gallbladder associated ectopic liver is the most common intra-abdominal location [10]. The reported sizes range from microscopic tissue to 3 cm [11]. The increased risk of hepatocellular carcinoma associated with ectopic liver tissue makes it an important anomaly that may pose a challenge to surgeons [8]. We present a case of ectopic liver attached to gall bladder serosa that was discovered incidentally during an elective laparoscopic cholecystectomy.

## 2. Case Report

The patient was a 42-year-old female with a known case of asthma. She had recurrent episodes of upper abdominal pain referred to back and right shoulder which was associated with fatty meals. Ultrasound showed multiple gall stones. She was admitted for elective laparoscopic cholecystectomy on January 8^th^ 2019.

**Figure 1:**
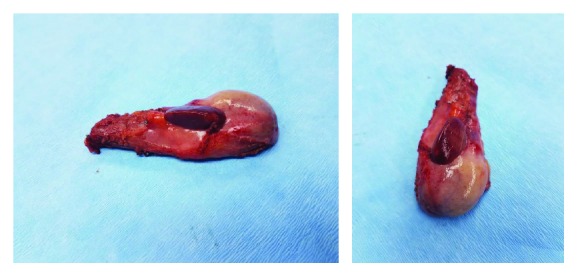
Gross pathology showing ectopic liver on the anterior surface of the gall bladder.

**Figure 2:**
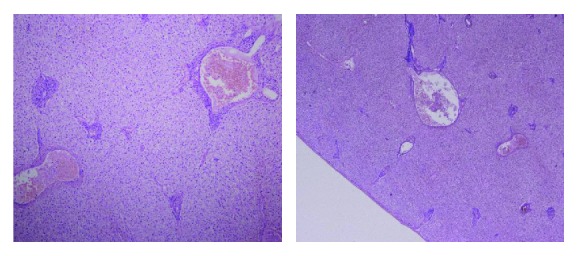
Histopathology report on ectopic liver tissue attached to the gall bladder showing normal lobular architecture.

Intraoperatively, there was a maroon-colored nodule attached to the anterior gall bladder wall as shown in Figure 1. En-bloc resection along with the gall bladder was done. Postoperatively, the patient stayed at the hospital for one day and was then discharged home. The resected specimen was sent to the histopathology department, and the report showed normal lobular architecture as shown in Figure 2.

## 3. Discussion

The incidence for ectopic liver tissue is significantly low with a reported prevalence of 0.47% [12]. There are several theories which exist to explain the presence of ectopic liver [13]. However, it is largely believed to develop during the fourth week in utero during the embryonic development of the liver, which occurs as a result of the displacement of a portion of the cranial part of the hepatic diverticulum of the liver bud to other sites [1, 3]. The ectopic liver is usually attached to the serosa of the gallbladder or within its wall, and it may also be found in the lumen of the gallbladder [7].

Ectopic tissue should be removed because it is predisposed to developing neoplastic transformation [6] regardless of the mother liver. Most reported cases of hepatocellular carcinoma in ectopic liver have been reported from Japan [6]. It is speculated that the ectopic tissue is more likely to develop into a malignancy due to the lack of a complete vasculature or ductal system and hence the possibility of it being functionally impaired. Chronic inflammation or cirrhosis can result from altered hepatic function, which increases the risk of carcinoma [14].

The diagnosis of ectopic liver tissue usually occurs when the patient has other medical conditions such as gall bladder stones or other biliary disease. Usually it is an incidental finding intraoperatively with its diagnosis preoperatively being extremely rare [15].

Typically, the patient is asymptomatic but in rare situations symptoms might occur such as upper abdominal pain due to torsion, hemorrhagic necrosis, rupture, or some form of compression induced by the mass due to malignant transformation to hepatocellular carcinoma.

The most common site of ectopic liver tissue is the gall bladder although other locations reported include the adrenal gland, pancreas, spleen, falciform ligament, pylorus, umbilicus retroperitoneum, and pericardium [7].

Ectopic liver tissue does not contain complete physiological hepatic lobule architecture and often lacks a complete vascular and ductal system, which facilitates the carcinogenic process. Hence, en-bloc removal is advised.

## 4. Conclusion

Ectopic liver tissue is a rare entity, usually diagnosed intraoperatively. The most common place for ectopic liver tissue is the gall bladder. The pathophysiology is still not clearly understood, and the management of choice is en-bloc resection due to the association of malignancy or ectopic liver tissue torsion.
